# Analysis of reproductive behavior in male pigeons in the White Mirthys and Egyptian Baladi breeds injected with testosterone propionate

**DOI:** 10.1186/s12917-026-05854-5

**Published:** 2026-09-05

**Authors:** Omar A. Mohamed, Albert R. Reyad, Alaa E. Elkomy, Ibrahim M. Khattab

**Affiliations:** 1 Department of Poultry Production, Faculty of Desert and Environmental Agriculture, Matrouh University, Matrouh, Egypt; 2 Livestock Research Department, Arid Lands Cultivation Research Institute, City of Scientific Research and Technological Applications (SRTA-City), New Borg El Arab, 21934 Egypt; 3 Department of Animal and Fish Production, Faculty of Desert and Environmental Agriculture, Matrouh University, Matrouh, Egypt

**Keywords:** Domestic pigeon, Testosterone, Reproductive behavior, Reproductive performance, Squab mortality

## Abstract

**Background:**

In pigeons, reproductive success is driven primarily by male courtship and copulatory behavior, both of which require threshold levels of circulating testosterone. This study investigated the effects of exogenous testosterone propionate (TP) injection on serum testosterone levels, aggressive behaviors, sexual displays, nest defense, parental care, reproductive cycles, and squab mortality in two pigeon breeds (*Columba livia*): White Mirthys and Egyptian Baladi.

**Methods:**

A total of 27 adult pairs of White Mirthys pigeons and 27 adult pairs of Egyptian Baladi pigeons were randomly assigned to three experimental groups (*n* = 9 pairs per breed per group; total *N* = 54 cages). Treatments consisted of male-directed subcutaneous injections of either a vehicle control (CON; ethanol and sesame oil mixture) or TP at doses of 1 mg/kg (T1) or 2 mg/kg of body weight (T2).

**Results:**

Baseline serum testosterone was 47.9% higher in White Mirthys than Egyptian Baladi pigeons (*P* < 0.001). Repeated-measures analysis showed a significant Time×Treatment interaction (*P* < 0.001) as TP induced dose-dependent elevations in both lineages. TP heightened aggression; female and squab pecking increased uniformly (*P* < 0.001), while a significant Breed×Treatment interaction (*P* < 0.001) governed squab predation, which surged exclusively in White Mirthys high-dose groups but remained suppressed in Baladi. For sexual behavior, strutting showed a significant interaction (*P* = 0.003), suppressing earlier in White Mirthys (T1) before converging at T2, whereas vocal nesting and courtship feeding followed identical quadratic trajectories (*P* < 0.001). Brood care feeding exhibited a significant interaction (*P* < 0.001); TP entirely abolished the Baladi breed’s superior baseline, suppressing provisioning to a near-zero floor across both lines. Defense metrics plateaued uniformly (*P* < 0.001). Offspring survival was compromised via a significant Breed×Treatment interaction (*P* = 0.002); mortality rose from 5.6% (CON) to 15.2% (T2), peaking at 23.1% in White Mirthys due to neglect and aggression, but remained insulated at 5.6% in Baladi. The hatching-to-laying interval shortened exclusively in Baladi T2 pairs to 25.6 days (*P* = 0.021).

**Conclusions:**

In conclusion, exogenous testosterone propionate administration was associated with increased nest defense but reduced visual courtship, suppressed paternal provisioning, and higher squab mortality. Furthermore, these behavioral shifts were significantly modulated by breed background, with the White Mirthys lineage demonstrating marked vulnerability compared to the insulated resilience of the native Egyptian Baladi breed.

## Background

Avian reproduction and parental care are complex, highly coordinated systems governed by an intricate balance of endocrine mechanisms, environmental cues, and genetic factors [1]. In columbids, such as pigeons, reproductive success relies heavily on biparental investment. Unlike many other avian families, both male and female pigeons share intensive nesting duties, including egg incubation, territorial defense, and the specialized production of crop milk to feed vulnerable squabs [2, 3]. This intense cooperation requires the precise temporal suppression of territorial aggression in favor of courtship, cooperative nest building, and nurturing parental behaviors [4]. Due to their predictable breeding rhythms, pigeons have emerged as a premier model organism for investigating avian behavioral ecology and the neuroendocrine mechanisms of reproduction [5].

The endocrine system plays a central role in modulating these behavioral transitions. Testosterone, the primary androgen driving male reproductive behaviors, facilitates activities such as sexual interaction, aggression, and initial reproductive behaviors [6]. As a critical sex hormone, it influences the development of courtship and aggressive behaviors, alongside the formation of secondary sexual characteristics [7, 8]. While baseline testosterone levels are essential for initializing courtship and establishing territories early in the reproductive cycle, their prolonged elevation is widely hypothesized to conflict with later parental stages [9]. Elevated circulating testosterone levels promote social and territorial aggression but are fundamentally incompatible with high-investment parental care, such as nest attendance and feeding [10].

Physiological and behavioral responses to hormonal shifts are not uniform, being heavily influenced by breed-specific genetic architectures [11]. Selectively bred pigeon lineages, such as the White Mirthys and the Egyptian Baladi, exhibit distinct phenotypic traits, behavioral temperaments, and physiological baselines. The White Mirthys is an intensively selected, commercial utility breed characterized by rapid growth rates, high fertility, and accelerated reproductive cycles aimed at maximizing squab biomass production. Conversely, the local Egyptian Baladi breed represents a rustic, naturally adapted lineage known for its environmental resilience, traditional foraging behaviors, and distinct, slower reproductive performance. Understanding how these distinct genetic backgrounds interact with exogenous hormonal stressors is vital for clarifying the physiological limits and evolutionary trade-offs inherent in columbid reproductive management [12].

A critical knowledge gap remains regarding the precise dose-dependent threshold at which testosterone transitions from stimulating courtship to triggering pathological, maladaptive aggression in domestic columbids. While literature broadly documents that elevated androgens suppress paternal care in passerines, the impact of varying exogenous testosterone concentrations on the unique, crop-milk-dependent parental care systems of domestic pigeons remains unknown. This provides a critical framework to optimize breeding management, mitigate behavioral pathologies, and enhance production sustainability in columbid husbandry. Furthermore, existing research has largely ignored how breed-specific genetic architectures modulate these endocrine-driven behavioral switches. Specifically, it is unclear whether local domestic breeds, shaped by distinct artificial selection pressures for temperament and reproductive fitness, exhibit divergent sensitivities to identical hormonal stressors. Consequently, this study systematically evaluated the effects of two doses of exogenous testosterone propionate (TP) on behavioral shifts in male pigeons, ranging from courtship to aberrant actions such as squab predation, and assessed their links to life-history outcomes, including mortality rate and reproductive cycle duration.

## Materials and methods

### Experimental design

Twenty-seven adult pairs of White Mirthys pigeons (mean body weight: 505 ± 7.26 g) and 27 adult pairs of Egyptian Baladi pigeons (mean body weight: 415 ± 6.01 g) were randomly assigned to three experimental treatment groups, yielding 9 cages per breed × treatment group (total *N* = 54 independent breeding cages). To evaluate the effects of exogenous hyper-androgenism, adult male pigeons received localized subcutaneous injections administered into the loose skin at the base of the neck. The treatments consisted of a vehicle control group (CON), a low-dose TP group (T1), and a high-dose TP group (T2), receiving 0, 1, and 2 mg/kg of body weight, respectively.

Each treated male received a total of two sequential injections of TP (Testosterone Propionate 250 mg injection, India). Each dose was prepared and administered in a standardized 0.2 mL vehicle solution consisting of an ethanol–sesame oil mixture. To control for injection-related stress and vehicle effects, males in the CON group received 0.2 mL of the vehicle alone by subcutaneous injection. The injection schedule was synchronized with the reproductive cycle of each cage: the first injection was administered on the day the squabs hatched, and the second was administered five days later.

Due to a paucity of baseline literature establishing empirical dose-response curves for exogenous androgens in domestic columbids, these specific concentrations were adapted from validated protocols in commercial broiler chickens [13]. Broiler chickens provide a relevant physiological baseline because, similar to high-performance utility pigeon breeds such as the White Mirthys, both have been selectively bred for accelerated growth velocities and high metabolic throughput, which fundamentally alters baseline endocrine sensitivities. A dual-dose framework was intentionally implemented to investigate whether exogenous androgens induce a linear behavioral response or a threshold-gated physiological switch in domestic pigeons. By administering 1 mg/kg BW as a conservative low dose and 2 mg/kg BW as an elevated dose, this study sought to establish a clear comparative baseline for androgen sensitivity across structurally divergent avian production lineages.

### Birds management

Each pair of parent pigeons consisted of a male and a female. They were raised as independent families in metal cages that measured 60 × 60 × 50 cm. Each cage contained two nests, allowing the pair to raise their squabs and maintain a regular breeding cycle [3]. The cages were arranged in an open-house system, which ensured good hygiene and adequate natural ventilation. To provide sufficient illumination, artificial lighting was used to maintain 17 h of light per day [3]. Pigeons were fed a pellet diet twice daily, with feed and water provided ad libitum throughout the 45-day experimental period. The ingredients and composition of the diet are shown in Table 1.

**Table 1:** Ingredients and composition of the diet

Item	Diet
Ingredients, %	
Yellow corn	51.55
Soybean meal	18.00
Sorghum grain	11.27
Wheat bran	8.20
Corn gluten	5.00
Limestone	3.23
Dicalcium phosphate	1.80
NaCl	0.40
Premix^1^	0.30
Choline	0.10
DL-methionine	0.10
L-Lysine	0.05
Calculated analysis	
Metabolizable energy, kcal/kg	2838
Crude protein, %	17.12
Crude fiber, %	4.11
Ether extract, %	3.32
Calcium, %	1.57
Available phosphorus, %	0.48
Arginine, %	0.98
Methionine, %	0.41
Lysine, %	0.76
Total sulfur amino acids, %	0.71

^1^ Each kg of vitamin and mineral mixture contains: 12 M IU of vitamin A, 5 M IU of vitamin D3, 80,000 mg of vitamin E, 4,000 mg of vitamin K, 4,000 mg of vitamin B1, 9,000 mg of vitamin B2, 4,000 mg of vitamin B6, 20 mg of vitamin B12, 15,000 mg of pantothenic acid, 60,000 mg of nicotinic acid, 2,000 mg of folic acid, 150 mg of biotin, 400,000 mg of choline chloride, 15,000 mg of copper sulfate, 1,000 mg of calcium iodide, 40,000 mg of ferrous sulfate, 100,000 mg of manganese oxide, 100,000 mg of zinc oxide, and 300 mg of sodium selenite

### Testosterone assays

Blood samples were collected from the male pigeons on the day of squab hatching and again on post-hatching day 5. Blood was drawn from the wing vein into anticoagulant-free Vacutainer tubes and allowed to clot at room temperature. The samples were then centrifuged for 15 min at 1,500 × g, after which the serum was aspirated into 1.5 mL Eppendorf tubes and stored at − 20 °C until analysis. Serum total testosterone concentrations were quantified in duplicate using the automated second-generation Elecsys^®^ Testosterone II electrochemiluminescence immunoassay (ECLIA) kit (Cat. No. 05200067190; Roche Diagnostics, Mannheim, Germany). The system’s analytical measuring range was 0.025–15.0 ng/mL, with a minimum functional sensitivity threshold of 0.115 ng/mL. The platform demonstrated high analytical precision, with intra-assay and inter-assay coefficients of variation below 3.4% and 4.8%, respectively, across the experimental range.

### Behavioral observations and standardization

To facilitate consistent behavioral monitoring without introducing recording bias, all adult male pigeons across the experimental groups were marked weekly with a small spot of blue spray paint on the dorsal plumage. This method enabled clear individual identification during video analysis without revealing treatment assignments. To minimize observer effects and avoid disrupting the birds’ natural behavior, direct manual observations within the birds room were avoided. Instead, continuous digital monitoring was conducted using a synchronized four-camera EZVIZ smart-camera system permanently mounted 250 cm above the enclosures. The cameras were equipped with wide-angle lenses and strategically positioned to ensure that all 54 independent breeding cages remained fully visible without blind spots.

Behavioral recordings began on the first day of the brooding cycle (the day of squab hatching; Day 0) and were collected automatically during standardized two-hour sessions conducted daily from 9:00 PM to 11:00 PM throughout the experimental period. This nocturnal time window was selected because it captures sensitive nesting behaviors in columbids, including maternal incubation, male nest attendance, protection against nocturnal disturbances, and male-mediated nest defense. These behaviors are vital to squab survival and may be influenced by testosterone-driven aggression. Behavioral activities were quantified as the number of occurrences per breeding pair per hour. To avoid pseudoreplication, daily behavioral counts were averaged across observation days for each independent experimental unit, producing a single representative hourly occurrence rate per cage for final statistical modeling.

To eliminate observer bias and ensure comparability among groups, human-proximity assays were strictly standardized. These assays assessed male responses to human presence, including wing beating, body puffing, and active nest protection. They were conducted exclusively by a single primary investigator, who maintained a uniform walking speed (~ 0.5 m/s) and wore identical dark clothing during every session to minimize visual novelty. Each cage was tested exactly once during the daily observation window.

To maintain a rigorous double-blind framework, all archived video footage and proximity-test records were assigned randomized alphanumeric codes before behavioral quantification, thereby separating data interpretation from experimental group assignments. The video files were then scored using a comprehensive, predetermined behavioral ethogram. Baseline categories for sexual and nurturing behaviors were adapted from standardized columbid protocols [14], whereas definitions of pathological aggression, pecking, and defense were developed specifically for this experiment to capture the clinical outcomes of testosterone administration. Two trained behavioral observers independently cross-scored all video recordings while remaining fully blinded to cage treatment status. Inter-observer reliability was assessed using Cohen’s kappa coefficient and yielded excellent agreement (κ = 0.88), confirming strong scoring consistency.

To monitor the health and welfare of the adult pairs and their offspring throughout the trial, a systematic clinical assessment protocol was established. Visual health checks were conducted daily during morning maintenance routines (between 08:00 AM and 10:00 AM) to assess localized injuries, behavioral indicators of severe distress, and clinical signs of weakness. Adult birds and squabs were weighed weekly using a digital precision scale to track growth and identify potential maternal or paternal nutritional deprivation. In accordance with institutional animal welfare guidelines, an individual animal was removed from the study if it exhibited a cumulative 15% reduction in baseline body weight, severe physical trauma, or systemic weakness that did not respond to clinical intervention. Deceased squabs were collected immediately upon discovery and subjected to standard post-mortem macroscopic examinations.

These quantified behaviors were then classified into three primary functional categories:

#### Aggressive behaviors

- Pecking female: The male directs sharp, aggressive pecks at the female (dam).
- Pecking squabs: The male directs targeted, hostile pecks at the squabs.
- Wing beating: The male vigorously flaps its wings as an overt display of aggression toward a human observer approaching the cage.
- Squab predation: Severe, hyper-aggressive attacks targeting the squabs that resulted in direct physical injuries or mortality.

#### Male sexual behaviors

- Nest sitting accompanied by cooing: The male occupies the nest bowl while emitting characteristic vocalizations to reinforce the pair bond or signal nest ownership.
- Courtship feeding: The male attempts to entice and solicit the female by offering feed or simulating regurgitation.
- Strutting: The male exhibits a characteristic upright posture, puffing its chest and walking with exaggerated steps to display a desire to mate.

#### Nest defense and squab care behaviors

- Body puffing: A defensive, static posture where the male erects its feathers to protect the nest and deter an approaching human.
- Nest protection: The male actively pecks at an approaching human hand or observer to physically defend the nest area and squabs.
- Feeding squabs: The male actively regurgitates crop milk or crop contents to provision the squabs.

### Reproductive performance

Egg Laying Cycle: The intervals between egg layings were calculated in days, following the approach described by Mohamed et al. [3]. Period Between Hatching and Next Laying: The intervals from successful hatching to the next laying were calculated in days.

### Statistical analysis

All statistical analyses were performed using SPSS statistical software (Version 26; IBM Corp., Armonk, NY, USA). The individual breeding cage, containing one adult male, one adult female, and their squabs *n* = 9 cages per breed × treatment group; total *N* = 54 cages, was designated as the primary experimental unit. Behavioral activities were recorded as the number of occurrences per breeding pair per hour during a fixed daily two-hour observation period (9 p.m. to 11 p.m) throughout the experimental period. Daily behavioral counts were summarized by calculating the mean hourly occurrence rate for each experimental unit across the observation days to obtain a single representative value per unit. This approach minimized pseudoreplication by ensuring that statistical inference was based strictly on independent experimental units.

Prior to executing parametric analyses, data distributions were verified for normality using the Shapiro–Wilk test, and homogeneity of variance was assessed using Levene’s test. Statistical methods were tailored to the specific mathematical structure of each variable as follows: Serum testosterone levels were analyzed using a two-way mixed-model Repeated-Measures Analysis of Variance (RM-ANOVA). Time (Baseline vs. Post-treatment) was specified as the within-subject repeated factor, while Breed (White Mirthys vs. Egyptian Baladi) and Treatment (CON, T1, and T2) were designated as between-subject factors. This model evaluated main effects alongside the critical Time × Treatment and Time × Breed interaction terms to isolate individual baseline changes over time.

Normally distributed variables, including egg-laying cycle and hatching-to-laying intervals, were analyzed using a two-way General Linear Model (GLM) to evaluate the main effects of breed, treatment, and their two-way interaction (Breed × Treatment). Because behavioral activities (e.g., pecking, wing beating, body puffing, nest protection, courtship feeding, strutting, and squab feeding) represented count data characterized by low baseline values or zero-inflation, standard linear models were inappropriate. These metrics were analyzed via Generalized Linear Models (GZLM) utilizing a Poisson log-linear response or a Negative Binomial distribution, selected per behavior based on the Akaike Information Criterion (AIC) for optimal goodness-of-fit. Squab mortality rates were analyzed using a GZLM with a Binomial logistic regression distribution. The model evaluated the actual numbers of dead and surviving squabs relative to the total number at risk within each experimental unit.

For all models, when a significant main effect or interaction was detected (*P* < 0.05), post-hoc pairwise multiple comparisons were conducted using Tukey’s Honestly Significant Difference (HSD) post-hoc test or Wald chi-square pairwise decompositions to isolate differences among the six explicit breed × treatment groups. Statistical significance was defined as *P* < 0.05. Effect sizes for parametric analyses were quantified and reported as partial eta squared.

## Results

### Serum testosterone levels

The serum testosterone profiles of male White Mirthys and Egyptian Baladi pigeons are summarized in Table 2. Before hormone administration, the two genotypes differed significantly in baseline testosterone concentration (*P* < 0.001), with White Mirthys males exhibiting levels 47.9% higher than those of Egyptian Baladi males.

**Table 2:** Serum testosterone levels (ng/mL) in male White Mirthys and Egyptian Baladi pigeons before and after subcutaneous injection with testosterone propionate

Breed	Treatment	*n*	Baseline (before)	Post-treatment (after)	Change (Δ)
White Mirthys	CON	9	3.58 ± 0.18^a^	3.55 ± 0.17^b^	-0.03
	T1	9	3.57 ± 0.20^a^	3.95 ± 0.19^ab^	+ 0.38
	T2	9	3.59 ± 0.19^a^	4.41 ± 0.22^a^	+ 0.82
Breed main effect	Pooled	27	3.58 ± 0.19	3.97 ± 0.18	+ 0.39
Egyptian Baladi	CON	9	2.42 ± 0.15^b^	2.37 ± 0.14^c^	-0.05
	T1	9	2.44 ± 0.16^b^	3.13 ± 0.18^b^	+ 0.69
	T2	9	2.43 ± 0.17^b^	3.39 ± 0.20^b^	+ 0.96
Breed main effect	Pooled	27	2.43 ± 0.16	2.96 ± 0.17	+ 0.53
Treatment main effects	CON	18	3.00 ± 0.17	2.96 ± 0.16^c^	-0.04
(Pooled across breeds)	T1	18	3.01 ± 0.18	3.54 ± 0.19^b^	+ 0.53
	T2	18	3.01 ± 0.18	3.90 ± 0.21^a^	+ 0.89
Two-way ANOVA *P*-values	Breed (B)		< 0.001	< 0.001	-
(Analyzed per time point)	Treatment (T)		0.985	< 0.001	
	B x T		0.998	0.042	
Repeated-measures *P*-values	Time (time-course effect)		-	-	< 0.001
	Time × T		-	-	< 0.001
	Time × B		-	-	0.145

Data were analyzed using a Repeated-Measures Mixed ANOVA framework where Time (Baseline vs. Post-Treatment) served as the within-subject factor, while Breed and Treatment served as between-subject factors

Values are presented as Mean ± Standard Error (SE) (*n* = 9 birds per sub-group; total *N* = 54)

Values represent subgroup means ± standard error of the mean (SEM) based on *n* = 9 independent cages per subclass (*N* = 54 pairs total)

CON: Vehicle-injected control group (received 0.2 mL subcutaneous injection of ethanol and sesame oil mixture without hormone); T1: 1 mg/kg body weight testosterone propionate; T2: 2 mg/kg body weight testosterone propionate

^a–e^ Means within a column sharing different superscript letters differ significantly (*P* < 0.05) based on Tukey’s Honestly Significant Difference (HSD) post-hoc test. Repeated-measures ANOVA parameters assess the individual within-subject change over time across the 5-day experimental window

Repeated-measures analysis revealed significant within-subject changes over time (*P* < 0.001) and a significant Time × Treatment interaction (*P* < 0.001), indicating that exogenous TP administration produced distinct, dose-dependent longitudinal increases in serum testosterone in both breeds. In the CON groups, testosterone levels remained statistically unchanged throughout the 5-day experimental period. In contrast, subcutaneous TP injection substantially increased circulating testosterone. In White Mirthys males, serum testosterone levels increased by 10.6% in the T1 group and 22.8% in the T2 group relative to their respective baseline values. The increase was more pronounced in Egyptian Baladi males, whose testosterone levels rose by 28.3% in the T1 group and 39.5% in the T2 group.

Cross-sectional two-way ANOVA of the post-treatment concentrations revealed significant main effects of both breed (*P* < 0.001) and hormone treatment (*P* < 0.001). Overall, White Mirthys males maintained higher post-injection testosterone concentrations than Egyptian Baladi males across all treatment groups (*P* < 0.001). A significant post-treatment Breed × Treatment interaction was also detected (*P* = 0.042), primarily because the magnitude of the post-injection increases varied relative to the baseline differences between breeds. Nevertheless, the overall longitudinal pattern of hormonal change did not differ fundamentally between the genotypes, as indicated by the non-significant Time × Breed interaction (*P* = 0.145).

### Aggressive behaviors and squab predation

The effects of breed, exogenous TP administration, and their interaction on aggressive behaviors and squab predation are presented in Table 3. Subcutaneous TP administration significantly increased male pigeon aggression. For both female-directed pecking and squab pecking, treatment had a significant dose-dependent main effect (*P* < 0.001), with aggressive pecking reaching its highest levels under the high-dose treatment. Likewise, squab-pecking frequencies were highest in the T2 treatment group. The main effect of breed was non-significant for female-directed pecking (*P* = 0.784) and showed only a statistical trend for squab pecking (*P* = 0.092). Consequently, the Breed × Treatment interactions for female pecking (*P* = 0.812) and squab pecking (*P* = 0.315) were non-significant.

**Table 3:** Effect of subcutaneous testosterone propionate injections on aggressive behaviors and squab predation in male pigeons (occurrences/hour)

Breed	Treatment	Pecking female	Pecking squabs	Wing beating	Squab predation
White Mirthys	CON	0.05 ± 0.01^c^	0.02 ± 0.00^c^	0.41 ± 0.03^d^	0.01 ± 0.00^b^
	T1	0.43 ± 0.04^b^	0.32 ± 0.03^b^	0.39 ± 0.04^d^	0.00 ± 0.00^b^
	T2	1.29 ± 0.11^a^	0.92 ± 0.08^a^	1.48 ± 0.12^b^	17.70 ± 1.45^a^
Breed main effect	Pooled	0.59 ± 0.05	0.42 ± 0.04	0.76 ± 0.06^b^	5.90 ± 0.48
Egyptian Baladi	CON	0.05 ± 0.01^c^	0.02 ± 0.00^c^	1.29 ± 0.09^c^	0.01 ± 0.00^b^
	T1	0.39 ± 0.03^b^	0.24 ± 0.02^b^	1.27 ± 0.10^c^	0.00 ± 0.00^b^
	T2	1.29 ± 0.10^a^	0.74 ± 0.06^a^	2.49 ± 0.18^a^	0.00 ± 0.00^b^
Breed main effect	Pooled	0.58 ± 0.05	0.34 ± 0.03	1.35 ± 0.10^a^	0.00 ± 0.00
Treatment main effects	CON	0.05 ± 0.01^c^	0.02 ± 0.00^c^	0.85 ± 0.06^b^	0.01 ± 0.00^b^
	T1	0.41 ± 0.04^b^	0.28 ± 0.03^b^	0.83 ± 0.07^b^	0.00 ± 0.00^b^
	T2	1.29 ± 0.11^a^	0.83 ± 0.07^a^	1.48 ± 0.11^a^	8.85 ± 0.72^a^
*P*-value	Breed (B)	0.784	0.092	< 0.001	< 0.001
	Treatment (T)	< 0.001	< 0.001	< 0.001	< 0.001
	B × T	0.812	0.315	0.004	< 0.001

Values for continuous variables are presented as Mean ± Standard Error (SE) (*n* = 9 cages per breed × treatment group; total *N* = 54)

CON: Vehicle-injected control group (received 0.2 mL subcutaneous injection of ethanol and sesame oil mixture without hormone); T1: 1 mg/kg body weight testosterone propionate; T2: 2 mg/kg body weight testosterone propionate

^a–d^ Means within the same column with different superscript letters differ significantly (*P* < 0.05) based on Tukey’s Honestly Significant Difference (HSD) post-hoc test. B × T represents the interaction effect between pigeon breed and hormone treatment

In contrast, wing beating and squab predation exhibited statistically significant Breed × Treatment interactions (*P* = 0.004 and *P* < 0.001, respectively). Wing beating also showed a significant main effect of breed (*P* < 0.001), with Egyptian Baladi males displaying substantially higher baseline frequencies in the CON group than White Mirthys males. Although the low-dose hormone treatment did not significantly affect wing-beating frequencies in either breed relative to its respective control, the high-dose treatment produced a sharp increase in these physical displays. This increase was more pronounced in the Egyptian Baladi T2 group, which showed a 93.0% rise above its baseline level.

The most divergent behavioral response was observed for squab predation, which also showed a significant interaction (*P* < 0.001). Squab predation was virtually absent in both breeds under the CON and T1 treatments. Under the high-dose treatment, the Egyptian Baladi males continued to show no squab predation, whereas White Mirthys males exhibited a substantial increase in predatory events (*P* < 0.05). This breed-specific response contributed to the significant overall main effect of breed (*P* < 0.001).

### Male sexual behaviors

The effects of breed, TP administration, and their interaction on male pigeon sexual and courtship behaviors are presented in Table 4. Exogenous TP injections markedly altered male sexual displays, with strutting behavior showing a significant interaction with genetic background. Specifically, strutting frequency exhibited a significant Breed × Treatment interaction (*P* = 0.003), along with an independent main effect of breed (*P* = 0.012). Although the CON groups of both lineages showed identical frequencies, TP administration produced distinct suppressive effects in the two genetic groups. Under the T1 treatment, strutting frequency declined sharply in White Mirthys males, whereas Egyptian Baladi males maintained a significantly higher, intermediate display rate (*P* < 0.05). Under T2, strutting frequencies converged and reached their lowest levels in both breeds. This pattern accounts for the overall significant main effect of treatment (*P* < 0.001).

**Table 4:** Effect of subcutaneous testosterone propionate injections on male sexual and courtship behaviors in pigeons (occurrences/hour)

Breed	Treatment	Sitting in nest with cooing	Courtship feeding	Strutting display
White Mirthys	CON	0.55 ± 0.04^b^	0.56 ± 0.04^b^	1.68 ± 0.12^a^
	T1	0.94 ± 0.07^a^	0.80 ± 0.06^a^	0.28 ± 0.02^c^
	T2	0.41 ± 0.03^b^	0.43 ± 0.03^b^	0.32 ± 0.03^c^
Breed main effect	Pooled	0.63 ± 0.05	0.60 ± 0.04	0.76 ± 0.06
Egyptian Baladi	CON	0.55 ± 0.04^b^	0.56 ± 0.05^b^	1.68 ± 0.14^a^
	T1	0.94 ± 0.08^a^	0.80 ± 0.07^a^	0.86 ± 0.07^b^
	T2	0.41 ± 0.03^b^	0.43 ± 0.03^b^	0.32 ± 0.03^c^
Breed main effect	Pooled	0.63 ± 0.05	0.60 ± 0.05	0.95 ± 0.08
Treatment main effects	CON	0.55 ± 0.04^b^	0.56 ± 0.04^b^	1.68 ± 0.13^a^
	T1	0.94 ± 0.08^a^	0.80 ± 0.07^a^	0.57 ± 0.05^b^
	T2	0.41 ± 0.03^b^	0.43 ± 0.03^b^	0.32 ± 0.03^c^
*P*-value	Breed (B)	0.999	0.999	0.012
	Treatment (T)	< 0.001	< 0.001	< 0.001
	B × T	0.999	0.999	0.003

Values for continuous variables are presented as Mean ± Standard Error (SE) (*n* = 9 cages per breed × treatment group; total *N* = 54)

CON: Vehicle-injected control group (received 0.2 mL subcutaneous injection of ethanol and sesame oil mixture without hormone); T1: 1 mg/kg body weight testosterone propionate; T2: 2 mg/kg body weight testosterone propionate

^a–c^ Means within the same column with different superscript letters differ significantly (*P* < 0.05) based on Tukey’s Honestly Significant Difference (HSD) post-hoc test. B × T represents the interaction effect between pigeon breed and hormone treatment

In contrast, sitting in the nest with cooing and courtship feeding showed neither significant Breed × Treatment interactions (*P* = 0.999 for both metrics) nor independent main effects of breed (*P* = 0.999). Because the subgroup means for these behaviors were mathematically identical between the White Mirthys and Egyptian Baladi lineages at all treatment levels, the behavioral responses were entirely uniform across breeds. Both traits were influenced exclusively by a significant main effect of treatment (*P* < 0.001). For sitting in the nest with cooing, the low-dose injection produced a significant change (*P* < 0.05); however, further escalation to the high dose caused a complete reversal in both breeds. Courtship feeding followed an identical quadratic pattern under the main effect of treatment: frequencies increased significantly from baseline to the T1 groups before declining significantly under the high-dose T2 treatment (*P* < 0.05).

### Nest defense and squab care behaviors

The effects of breed, TP administration, and their interaction on nest defense and squab-care behaviors are presented in Table 5. Exogenous TP administration significantly altered patterns of nest care and parental investment, shifting male behavior away from squab care and toward active nest defense. Offspring-provisioning frequency showed a highly significant Breed × Treatment interaction, as well as significant main effects of breed (*P* < 0.001) and treatment (*P* < 0.001). Under CON conditions, the two lineages differed markedly: Egyptian Baladi males exhibited substantially higher baseline feeding frequencies than White Mirthys males (*P* < 0.05). However, this breed-specific advantage was eliminated following exogenous TP administration. Feeding frequencies declined uniformly in both genotypes in the low-dose and high-dose groups. This consistent reduction under hyperandrogenic conditions indicates that, although Egyptian Baladi males display superior baseline parental provisioning, both lineages are similarly sensitive to testosterone-mediated suppression of offspring care.

**Table 5:** Effect of subcutaneous testosterone propionate injections on nest defense and squab care behaviors in male pigeons (occurrences/hour)

Breed	Treatment	Body puffing	Nest protection	Feeding squabs
White Mirthys	CON	0.33 ± 0.02^c^	0.31 ± 0.02^c^	0.68 ± 0.05^a^
	T1	1.05 ± 0.08^a^	0.98 ± 0.07^a^	0.02 ± 0.00^c^
	T2	1.05 ± 0.09^a^	0.98 ± 0.08^a^	0.02 ± 0.00^c^
Breed main effect	Pooled	0.81 ± 0.06^a^	0.76 ± 0.06^a^	0.24 ± 0.02^b^
Egyptian Baladi	CON	0.11 ± 0.01^d^	0.21 ± 0.02^c^	1.91 ± 0.14^a^
	T1	0.73 ± 0.05^b^	0.64 ± 0.05^b^	0.02 ± 0.00^c^
	T2	0.73 ± 0.06^b^	0.64 ± 0.05^b^	0.02 ± 0.00^c^
Breed main effect	Pooled	0.52 ± 0.04^b^	0.50 ± 0.04^b^	0.65 ± 0.05^a^
Treatment main effects	CON	0.22 ± 0.02^b^	0.26 ± 0.02^b^	1.30 ± 0.10^a^
	T1	0.89 ± 0.07^a^	0.81 ± 0.06^a^	0.02 ± 0.00^b^
	T2	0.89 ± 0.07^a^	0.81 ± 0.06^a^	0.02 ± 0.00^b^
*P*-values	Breed (B)	< 0.001	0.002	< 0.001
	Treatment (T)	< 0.001	< 0.001	< 0.001
	B × T	0.658	0.412	< 0.001

Values for continuous variables are presented as Mean ± Standard Error (SE) (*n* = 9 cages per breed × treatment group; total *N* = 54)

CON: Vehicle-injected control group (received 0.2 mL subcutaneous injection of ethanol and sesame oil mixture without hormone); T1: 1 mg/kg body weight testosterone propionate; T2: 2 mg/kg body weight testosterone propionate

^a–d^ Means within the same column with different superscript letters differ significantly (*P* < 0.05) based on Tukey’s Honestly Significant Difference (HSD) post-hoc test. B × T represents the interaction effect between pigeon breed and hormone treatment

In contrast, body-puffing displays and active nest protection showed no significant Breed × Treatment interactions (*P* = 0.658 and *P* = 0.412, respectively), indicating that both lineages followed comparable behavioral trajectories across treatment doses. Nevertheless, both behaviors were influenced by highly significant independent main effects of breed (*P* < 0.001 for body puffing; *P* = 0.002 for nest protection) and treatment (*P* < 0.001 for both traits). White Mirthys males consistently displayed body puffing more frequently than Egyptian Baladi males across all treatment levels (*P* < 0.05), resulting in a higher pooled breed main effect. TP administration increased body-puffing frequency significantly from control levels to an elevated plateau in the T1 groups, with no further change in the high-dose T2 groups (*P* < 0.05). Nest protection followed a similar treatment-related trajectory, increasing significantly from control levels to a stable, elevated threshold in both the T1 and T2 groups (*P* < 0.05).

### Reproductive performance

The effects of breed, TP administration, and their interaction on female reproductive cycles and offspring survival are presented in Table 6. Exogenous TP injections compromised offspring survival through a significant Breed × Treatment interaction (*P* = 0.002) but did not significantly disrupt the overall egg-laying cycle (*P* = 0.415).

**Table 6:** Effect of subcutaneous testosterone propionate injections on female reproductive cycles and offspring mortality rates in pigeons

Breed	Treatment	Egg laying cycle (days)	Hatching-to-laying (days)	Squab mortality rate (%)
White Mirthys	CON	45.2 ± 3.1	30.4 ± 2.1	5.6% (1/18)^c^
	T1	47.1 ± 3.5	31.2 ± 2.4	11.1% (2/17)^b^
	T2	46.9 ± 3.4	31.4 ± 2.2	23.1% (4/16)^a^
Breed main effect	Pooled	46.4 ± 3.3	31.0 ± 2.2	13.7% (7/51)^a^
Egyptian Baladi	CON	45.0 ± 2.9	30.0 ± 1.9	5.6% (1/18)^c^
	T1	47.5 ± 3.6	30.8 ± 2.1	6.7% (1/15)^bc^
	T2	46.5 ± 3.2	25.6 ± 1.8	5.9% (1/17)^c^
Breed main effect	Pooled	46.3 ± 3.2	28.8 ± 1.9	6.0% (3/50)^b^
Treatment main effects	CON	45.1 ± 3.0	30.2 ± 2.0	5.6% (2/36)^c^
	T1	47.3 ± 3.5	31.0 ± 2.2	9.4% (3/32)^b^
	T2	46.7 ± 3.3	28.5 ± 2.0	15.2% (5/33)^a^
*P*-value	Breed (B)	0.942	0.015	< 0.001
	Treatment (T)	0.415	0.041	< 0.001
	B × T	0.887	0.021	0.002

Values for continuous variables are presented as Mean ± Standard Error (SE) (*n* = 9 cages per breed × treatment group; total *N* = 54)

Continuous variables were verified via Shapiro-Wilk and Levene’s tests. Proportional squab mortality was evaluated using a Generalized Linear Model (GZLM) with a binomial logistics distribution based on actual numbers of surviving and dead squabs at risk

CON: Vehicle-injected control group (received 0.2 mL subcutaneous injection of ethanol and sesame oil mixture without hormone); T1: 1 mg/kg body weight testosterone propionate; T2: 2 mg/kg body weight testosterone propionate

^a–c^ Means or percentages within a column sharing different superscript letters differ significantly (*P* < 0.05) based on post-hoc pairwise Wald chi-square or Duncan’s multiple range tests. B × T represents the interaction effect between pigeon breed and hormone treatment

Squab mortality rates showed significant Breed × Treatment interaction (*P* = 0.002) as well as prominent independent main effects for breed (*P* < 0.001) and treatment (*P* < 0.001). Under CON conditions, both genetic lineages displayed the same baseline squab mortality rate of 5.6%. Following male TP administration, pooled mortality increased in a dose-dependent manner, rising to 9.4% in the T1 group and peaking at 15.2% in the T2 treatment group (*P* < 0.05). However, the two genotypes diverged markedly under the highest androgen dose. The White Mirthys lineage was particularly vulnerable to offspring loss, with squab mortality increasing to 11.1% under the T1 dose and reaching 23.1% in the high-dose T2 treatment group. In contrast, the native Egyptian Baladi breed showed no comparable increase in mortality, maintaining low rates of 6.7% in the T1 group and 5.6% under the maximum T2 dose (*P* > 0.05).

In contrast to these survival outcomes, male hormone treatment did not significantly alter the primary female egg-laying cycle. Neither the main effects of breed and treatment nor their interaction were significant for this measure (breed: *P* = 0.942; treatment: *P* = 0.415; interaction: *P* = 0.887), with values remaining relatively consistent at approximately 45–47 days across all treatment groups. However, the hatching-to-laying interval exhibited significant main effects of breed (*P* = 0.015) and treatment (*P* = 0.041), as well as a significant Breed × Treatment interaction (*P* = 0.021). Although this interval remained statistically unchanged or increased slightly under testosterone treatment in White Mirthys pairs, it decreased sharply in the high-dose Egyptian Baladi group (*P* < 0.05).

## Discussion

### Effect of TP injections on serum testosterone levels

Baseline evaluations of serum testosterone revealed no significant differences among the designated treatment groups within either breed. This absence of baseline variation confirms effective randomization across experimental units and ensures that subsequent post-injection physiological and behavioral changes can be attributed directly to the exogenous treatments rather than to pre-existing hormonal differences. However, a distinct baseline difference was observed between the two genotypes: White Mirthys pigeons exhibited inherently higher serum testosterone levels than their Egyptian Baladi counterparts. This divergence may reflect fundamental genetic and physiological differences between the two domestic lineages. In avian models, baseline androgen levels often reflect life-history strategies or the effects of artificial selection. Populations bred for specific behavioral traits, territoriality, or localized performance characteristics may therefore maintain distinct patterns of circulating androgen regulation [15, 16].

Following the injection of TP, serum testosterone profiles changed markedly in both breeds. Exogenous administration successfully increased circulating testosterone levels in a dose-dependent manner. Whereas the CON group maintained relatively stable baseline concentrations, the low-dose (T1; 1 mg/kg) treatment increased the pooled post-treatment concentration to 3.54 ng/mL. The greatest increase occurred in the high-dose (T2; 2 mg/kg) group, in which the overall post-treatment testosterone concentration reached 3.90 ng/mL, indicating a clear hyperandrogenic state. This predictable dose-related increase confirms the efficacy of subcutaneous TP as a reliable method for elevating systemic androgen concentrations in columbid species and is consistent with established avian models that use steroid administration to simulate circulating androgen peaks [17–19].

The repeated-measures design enabled a more robust assessment of individual changes over time. The significant Time × Treatment interaction confirms that the longitudinal changes in serum testosterone were driven by hormone dose. Although the cross-sectional analysis of the post-treatment phase revealed a significant Breed × Treatment interaction, this finding primarily reflected the higher absolute post-injection testosterone concentrations maintained by White Mirthys pigeons because of their elevated baseline levels. In contrast, the non-significant Time × Breed interaction indicates that physiological responsiveness to exogenous testosterone propionate, including the relative rate of change and systemic accumulation of the hormone, was comparable and proportional across the two genetic backgrounds.

### Effect of TP injections on aggressive behaviors and squab predation

The escalation in male aggression following high-dose testosterone administration provides a behavioral link to the observed increase in offspring mortality. These findings are consistent with earlier investigations documenting positive associations between elevated androgen concentrations and agonistic interactions in various avian species [20–22]. This behavioral pattern was accompanied by a dose-dependent increase in serum testosterone, which peaked at 3.90 ng/mL in the pooled T2 treatment group. Elevated peripheral hormone concentrations increase the volume of circulating steroids available to influence downstream neuroendocrine processing. Within the central nervous system, testosterone interacts with localized receptor pathways that regulate social behavior, parental motivation, and territorial defense [23, 24]. The marked increases in female and squab pecking under T2 treatment align with the mating–parenting trade-off hypothesis of avian reproduction, suggesting that elevated systemic androgen concentrations may shift behavior away from paternal investment and toward aggression against conspecifics [25, 26].

The emergence of squab predation, reflected in a significant Breed × Treatment interaction, indicates that genetic lineage influences behavioral sensitivity to similar hormonal thresholds. Although White Mirthys pigeons exhibited higher baseline and post-injection serum testosterone levels than Egyptian Baladi pigeons, the Egyptian Baladi strain maintained a higher baseline wing-beating frequency. This divergence demonstrates that behavioral responses are not a direct, linear function of circulating hormone concentrations; rather, genetic background strongly influences how peripheral hormonal surges are integrated. The observed variation could reflect unmeasured breed-specific differences in localized receptor density, central aromatase activity, or neural sensitivity to testosterone [27, 28]. Collectively, these findings suggest that increased squab mortality is associated with hyperandrogenism-induced paternal aggression and localized predatory behavior. However, because the central molecular and enzymatic mechanisms were not directly quantified in the current study, these physiological pathways remain tentative hypotheses requiring future empirical validation.

A distinct phenotypic divergence was evident in the significant main effect of breed on wing-beating frequency, with Egyptian Baladi pigeons displaying substantially higher overall activity than White Mirthys pigeons. This difference persisted across treatments, even though both breeds exhibited virtually identical baseline levels of female pecking, squab pecking, and squab predation in the CON groups. The partial dissociation of wing-beating from generalized testosterone-driven aggression suggests that this physical display represents a breed-specific characteristic rather than a shared neuroendocrine response. This divergence may reflect baseline physiological, metabolic, or physical differences [29–33] between the two genetic lineages. However, because potential mechanisms, including body-mass scaling, muscle-to-bone ratios, and locomotor adaptations, were not directly evaluated, these physical explanations remain unverified. Accordingly, this behavioral variation should be interpreted conservatively as an inherent breed trait that is modulated by, but independent of, generalized testosterone-mediated aggression. Future morphometric analyses could help clarify the physical basis of this behavior.

### Effect of TP injections on nest defense and squab care behaviors

The changes in paternal provisioning and defensive displays following testosterone administration provide a behavioral link to the observed differences in squab survival. The severe reduction in feeding frequency under TP treatments represents a substantial decline in parental investment, thereby increasing offspring vulnerability to starvation. This behavioral shift is consistent with the classic mating–parenting trade-off hypothesis, which proposes that elevated androgen levels are associated with the reallocation of finite energetic resources from offspring care toward territorial defense and other defensive behaviors, such as body puffing and nest protection. In columbids, these parental behaviors are closely linked to prolactin-mediated systems that regulate crop-milk production and delivery. Circulating androgens may act as central antagonists of prolactin pathways, potentially disrupting the neuroendocrine networks required to maintain regular regurgitation feeding while simultaneously modifying localized defensive responses [34, 35].

The significant Breed × Treatment interaction for squab-feeding frequency confirms that genetic background modulates the behavioral response of these lineages to hormonal shifts during the nesting phase. Under vehicle-control conditions, the native Egyptian Baladi breed demonstrated substantially greater baseline investment in offspring provisioning than the White Mirthys lineage. However, exogenous hyperandrogenism eliminated this genetic advantage, reducing squab provisioning to a uniform, near-zero level in both breeds under the T1 and T2 doses. This uniform decline indicates that although baseline investment differs between breeds, the neuroendocrine pathways regulating offspring provisioning are similarly sensitive to androgenic suppression in both domestic lineages.

In contrast, body puffing and nest-protection behaviors showed no significant Breed × Treatment interactions, indicating that both breeds followed comparable behavioral trajectories after TP injection. Both behaviors increased from their control baselines to a stable, elevated plateau under the treatment main effects. Nevertheless, White Mirthys males maintained significantly higher baseline and post-injection levels than Egyptian Baladi males across the corresponding groups. This divergence suggests that behavioral responses are not a direct, linear function of circulating hormone concentrations; rather, genetic background may influence how peripheral hormonal surges are integrated [36]. These differences may reflect breed-specific variation in neural receptor density or central hormone processing, although these mechanisms were not measured in the present study. Accordingly, these physiological explanations remain tentative and require empirical validation in future research.

### Effect of TP injections on nest defense and squab care behaviors

The changes in paternal provisioning and defensive displays following testosterone administration provide a behavioral link to the observed variations in squab survival. The reduction in feeding frequency under high-dose T2 treatment represents a substantial decline in parental investment, which can increase offspring vulnerability to starvation. This behavioral shift is consistent with the classic mating-parenting trade-off hypothesis, in which elevated androgens are associated with a reallocation of finite energetic resources away from offspring care and toward territorial defense, such as body puffing and nest protection [37–39]. In columbids, these behaviors are closely linked to prolactin-mediated systems that regulate crop milk production and delivery. Circulating androgens can operate as central antagonists to prolactin pathways, potentially altering the neural networks required to maintain regular regurgitation feeding while simultaneously modifying localized defensive responses.

The highly significant Breed × Treatment interactions for squab feeding frequency, body puffing, and active nest protection show that genetic background modulates how these lineages respond behaviorally to hormonal shifts. The behavioral outcomes reveal that under high-dose T2 hormone stress, the local Egyptian Baladi breed demonstrated a higher commitment to offspring provisioning and active defense, with body puffing displays increasing to their highest recorded levels. Conversely, the White Mirthys lineage displayed a severe behavioral decline under the same T2 dose, with nest protection collapsing and squab feeding dropping. This divergence suggests that the two breeds process peripheral testosterone surges through distinct thresholds. These variations are hypothesized to reflect potential, though unmeasured, breed-specific configurations in neural receptor density or central hormone processing. Because these localized endocrine mechanisms were not directly assayed in this study, these pathways remain tentative hypotheses requiring future empirical validation.

### Effect of TP injections on reproductive performance

The increase in squab mortality following paternal TP administration represents a clear life-history cost. Overall mortality increased in a dose-dependent manner, rising from 5.6% in the CON group to 15.2% in the pooled high-dose T2 group. This increase was accompanied by distinct behavioral and physiological changes. Rather than reflecting an indirect shift in macro-reproductive timing, the mortality trend appears to have resulted directly from the behavioral disruptions observed across the treatment cohorts. Under the low-dose treatment, squab losses coincided with a marked reduction in paternal provisioning, which subjected squabs to nutrient deprivation. Under the high-dose treatment, this parental neglect was compounded by active paternal aggression, including vigorous pecking.

To systematically document these survival outcomes, clinical assessments were conducted. Affected squabs exhibited symptoms of acute malnutrition, including a progressive loss of body weight and severe clinical debilitation. Moreover, these chicks endured direct paternal aggression, males inflicted physical trauma, primarily localized to the head and dorsal regions, through sharp, vigorous pecking that resulted in visible tissue hemorrhaging and localized feather plucking. Post-mortem macro-examinations of the deceased chicks confirmed the absence of internal lesions or clinical signs attributable to endemic disease or systemic infection, indicating that trauma and starvation were the primary drivers of mortality. This pattern reinforces the hypothesis that hyper-androgenism reduces columbid reproductive success by disrupting essential paternal care pathways and increasing conspecific aggression toward vulnerable offspring.

The significant Breed × Treatment interaction for squab mortality, evaluated using a binomial logistic regression model, indicates that genetic background strongly modulates offspring susceptibility to paternal hormonal surges. Although both lineages initially exhibited increased mortality under the low-dose T1 condition, their responses diverged under the high-dose T2 intervention. The White Mirthys lineage showed pronounced vulnerability to offspring loss under maximum androgen exposure, with squab mortality reaching 23.1% in the T2 group. This elevated mortality was closely associated with low baseline provisioning and a subsequent decline in effective nest defense under high hormonal exposure, leaving the brood vulnerable to both parental neglect and direct paternal aggression. In contrast, the native Egyptian Baladi breed remained below this lethal threshold, maintaining a low squab mortality rate of 5.6% under the maximum T2 dose. This resilience may reflect their stronger baseline commitment to squab care and greater behavioral stability under hyperandrogenic conditions.

Breed-specific variation was also evident in the hatching-to-laying intervals, which showed a significant Breed × Treatment interaction. Whereas the reproductive interval of White Mirthys pairs remained unchanged, Egyptian Baladi pairs significantly shortened their hatching-to-laying interval under the T2 dose. This accelerated reproductive cycling suggests that Egyptian Baladi pairs may employ an adaptive compensatory strategy to offset treatment-induced changes in male behavior by adjusting their inter-clutch intervals and thereby maintaining overall fitness. However, because central receptor densities, metabolic scaling, and prolactin–androgen crosstalk were not directly quantified in the current study, these proposed physiological mechanisms remain tentative and require molecular validation in future research.

### Limitations and future studies

While this study provides clear evidence that testosterone injections influence aggression and parental care in pigeons, several key limitations must be acknowledged.

The experimental design utilized a relatively short daily observation window following testosterone propionate administration. While this approach effectively captured acute behavioral and physiological responses, it remains unclear whether these effects persist across the diurnal cycle. Furthermore, because behavioral data were averaged across observation days to avoid pseudoreplication, our temporal resolution was limited, constraining our ability to evaluate dynamic behavioral shifts over time. Future studies should incorporate extended observation windows and continuous longitudinal tracking to better characterize these temporal dynamics.

The study focused on a restricted profile of physiological markers. The exclusion of hormones such as oxytocin, prolactin, and glucocorticoids, well-documented modulators of parental behavior and bonding, prevents a definitive link between the observed outcomes and a comprehensive endocrine profile. Future research incorporating simultaneous profiling of testosterone, prolactin, and corticosterone, alongside receptor expression analyses, is warranted to better disentangle these interconnected neuroendocrine mechanisms.

A conceptual limitation of the current experimental design is that testosterone treatment was administered exclusively to male pigeons, while several key reproductive and parental outcomes were evaluated at the pair or family level. Consequently, we cannot rule out the influence of an unavoidable biological confounding factor: female compensatory behavior. It is possible that unmeasured adjustments in the female partners’ parental investment or behavior indirectly affected or mitigated the observed family-level outcomes. Future studies should consider monitoring female behavioral responses or treating both partners to isolate these parental dynamics more precisely.

## Conclusion

In conclusion, this study demonstrates that exogenous TP administration was associated with a distinct reallocation of reproductive priorities in male pigeons, linking elevated circulating androgens to heightened territorial dominance and nest defense at the expense of parental investment and offspring survival. Although White Mirthys males possessed an inherently higher baseline of circulating testosterone than Egyptian Baladi males, both breeds exhibited clear, dose-dependent increases in serum testosterone levels post-injection. This hormonal elevation was linked to increased aggressive dominance, reflected in uniform increases in pecking directed toward females and squabs alongside enhanced nest-defense behaviors such as body puffing. Simultaneously, male hyper-androgenism was associated with a marked suppression of cooperative reproductive behaviors, including nest-sitting, courtship feeding, strutting, and squab provisioning. While this endocrine elevation did not alter the temporal pacing of the primary egg-laying cycle, it significantly altered hatching-to-laying intervals and offspring survival via strong breed-dependent interactions. High-dose treatments were associated with a severe spike in squab predation exclusively within the White Mirthys cohort. Conversely, the native Egyptian Baladi breed demonstrated greater behavioral stability under hyper-androgenic conditions.

## Data Availability

Datasets generated and/or analyzed during this study are included in this article version, and if required any further information related to the data involved in the manuscript can be obtained from the corresponding author upon reasonable request.

## Abbreviations

AIC: Akaike Information Criterion
ANOVA: Analysis of variance
BW: Body weight
CON: Vehicle control group (received 0.2 mL subcutaneous injection of ethanol and sesame oil mixture without hormone)
ELISA: Enzyme-linked immunosorbent assay
GLM: General linear model
GZLM: Generalized linear model
HPG: Hypothalamic-pituitary-gonadal axis
RM-ANOVA: Repeated-measures analysis of variance
SE: Standard error
T1: Low-dose testosterone propionate treatment group (1 mg/kg body weight)
T2: High-dose testosterone propionate treatment group (2 mg/kg body weight)
TP: Testosterone propionate
